# Survey on Non-Human Primates and Mosquitoes Does not Provide Evidences of Spillover/Spillback between the Urban and Sylvatic Cycles of Yellow Fever and Zika Viruses Following Severe Outbreaks in Southeast Brazil

**DOI:** 10.3390/v12040364

**Published:** 2020-03-26

**Authors:** Filipe Vieira Santos de Abreu, Anielly Ferreira-de-Brito, Adriana de Souza Azevedo, José Henrique Rezende Linhares, Vanessa de Oliveira Santos, Emily Hime Miranda, Maycon Sebastião Alberto Santos Neves, Lena Yousfi, Ieda Pereira Ribeiro, Alexandre Araújo Cunha dos Santos, Edmilson dos Santos, Taissa Pereira dos Santos, Danilo Simonini Teixeira, Marcelo Quintela Gomes, Camilla Bayma Fernandes, Andrea Marques Vieira da Silva, Monique da Rocha Queiroz Lima, Christophe Paupy, Alessandro Pecego Martins Romano, Ana Paula Dinis Ano Bom, Luzia Maria de Oliveira-Pinto, Sara Moutailler, Monique de Albuquerque Motta, Márcia Gonçalves Castro, Myrna Cristina Bonaldo, Sheila Maria Barbosa de Lima, Ricardo Lourenço-de-Oliveira

**Affiliations:** 1 Laboratório de Mosquitos Transmissores de Hematozoários, Instituto Oswaldo Cruz, Rio de Janeiro 21040-900, Brazil; filipe.vieira@ifnmg.edu.br (F.V.S.d.A.); aniellya@gmail.com (A.F.-d.-B.); mayconnev@gmail.com (M.S.A.S.N.); marcquintela@gmail.com (M.Q.G.); moniquemot@gmail.com (M.d.A.M.); marciadengue@gmail.com (M.G.C.); 2 Instituto Federal do Norte de Minas Gerais, Salinas 39560-000, Brazil; 3 Laboratório de Tecnologia Virológica, Instituto de Tecnologia em Imunobiológicos Bio-Manguinhos, Rio de Janeiro 21040-900, Brazil; adriana.soares@bio.fiocruz.br (A.d.S.A.); henrique.rznd@hotmail.com (J.H.R.L.); vanessa.oliveira@bio.fiocruz.br (V.d.O.S.); emily@bio.fiocruz.br (E.H.M.); smaria@bio.fiocruz.br (S.M.B.d.L.); 4 UMR BIPAR, Animal Health Laboratory, ANSES, INRA, Ecole Nationale Vétérinaire d’Alfort, Université Paris-Est, 94700 Maisons-Alfort, France; lenayousfi@hotmail.fr (L.Y.); sara.moutailler@anses.fr (S.M.); 5 Laboratório de Biologia Molecular de Flavivirus, Instituto Oswaldo Cruz, Rio de Janeiro 21040-900, Brazil; iedapr@gmail.com (I.P.R.); xande_acs@hotmail.com (A.A.C.d.S.); mbonaldo@ioc.fiocruz.br (M.C.B.); 6 Divisão de Vigilância Ambiental em Saúde, Secretaria de Saúde do Rio Grande do Sul, Porto Alegre 90610-000, Brazil; edmilsondvas@gmail.com; 7 MIVEGEC, CNRS, Institut de Recherche pour le Développement (IRD), Université de Montpellier, 34394 Montpellier, France; tayssadnz@gmail.com (T.P.d.S.); christophe.paupy@ird.fr (C.P.); 8 Núcleo de Atendimento e Pesquisa de Animais Silvestres, Universidade Estadual de Santa Cruz, Ilhéus 45662-900, Brazil; simonini.danilo@gmail.com; 9 Laboratório de Tecnologia Imunológica, Instituto de Tecnologia em Imunobiológicos Bio-Manguinhos, Rio de Janeiro 21040-900, Brazil; camilla.bayma@bio.fiocruz.br (C.B.F.); amarques@bio.fiocruz.br (A.M.V.d.S.); adinis@bio.fiocruz.br (A.P.D.A.B.); 10 Laboratório de Imunologia Viral, Instituto Oswaldo Cruz, Rio de Janeiro 21040-900, Brazil; moniquerql@gmail.com (M.d.R.Q.L.); lmopnogueira@gmail.com (L.M.d.O.-P.); 11 Secretaria de Vigilância em Saúde, Ministério da Saúde, Brasília 70719-040, Brazil; alessandropecego@gmail.com

**Keywords:** arboviruses, *Flavivirus*, serology, PRNT, high throughput real time PCR

## Abstract

In the last decade, Flaviviruses such as yellow fever (YFV) and Zika (ZIKV) have expanded their transmission areas. These viruses originated in Africa, where they exhibit both sylvatic and interhuman transmission cycles. In Brazil, the risk of YFV urbanization has grown, with the sylvatic transmission approaching the most densely populated metropolis, while concern about ZIKV spillback to a sylvatic cycle has risen. To investigate these health threats, we carried out extensive collections and arbovirus screening of 144 free-living, non-human primates (NHPs) and 5219 mosquitoes before, during, and after ZIKV and YFV outbreaks (2015–2018) in southeast Brazil. ZIKV infection was not detected in any NHP collected at any time. In contrast, current and previous YFV infections were detected in NHPs sampled between 2017 and 2018, but not before the onset of the YFV outbreak. Mosquito pools screened by high-throughput PCR were positive for YFV when captured in the wild and during the YFV outbreak, but were negative for 94 other arboviruses, including ZIKV, regardless of the time of collection. In conclusion, there was no evidence of YFV transmission in coastal southeast Brazil before the current outbreak, nor the spread or establishment of an independent sylvatic cycle of ZIKV or urban *Aedes aegypti* transmission of YFV in the region. In view of the region’s receptivity and vulnerability to arbovirus transmission, surveillance of NHPs and mosquitoes should be strengthened and continuous.

## 1. Introduction

In the last decade, several arboviruses of medical importance have caused outbreaks of global or national dimensions, among which are Zika (ZIKV) and Yellow Fever (YFV) viruses [1,2,3]. Both are *Flavivirus* originating from Africa, where they exhibit at least two ecologically distinct transmission cycles: a sylvatic cycle, in which arboreal mosquito species transmit them among non-human primates (NHPs), with humans accidentally infected; and an interhuman cycle, where the virus is transmitted among humans by *Aedes* mosquitoes, including the anthropophilic mosquito *Aedes aegypti* in the domicile or peridomicile [4,5,6,7]. The worldwide spread of the competent vector *Ae. aegypti* favored the dissemination of YFV and ZIKV out of Africa, triggering severe urban outbreaks in several continents, but at different times. 

**Figure 1:**
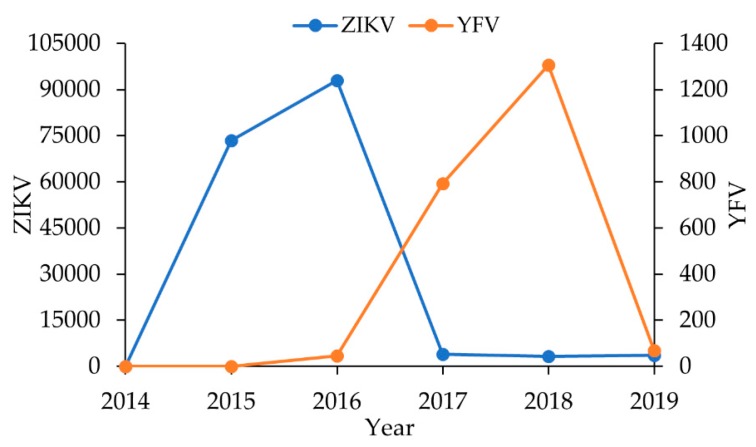
Number of human cases of Zika virus (ZIKV) and Yellow Fever virus (YFV) registered in Brazilian Southeast between 2014 and 2019 (https://www.saude.gov.br/boletins-epidemiologicos). In 2015, the year of the emergence of ZIKV in Brazil, there were no specific diagnostic and notification protocols. Therefore, the number of ZIKV cases showed in 2015 is an estimate for Brazilian Southeast, based in the conservative number of 440,000 cases in Brazil, in 2015, predicted by Helkeubach et al. (2016) [21].

Urban Yellow Fever has been identified in the Americas since the 17th century, causing great outbreaks, especially in port cities [8,9]. Until 1930, only the YFV urban transmission cycle was described [7,9] The establishment of a sylvatic cycle in the New World was documented after confirmation of human cases occurred in an *Ae. aegypti*-free rural area in southeast Brazil in 1932 [10]. It was subsequently shown that YFV had adapted to neotropical NHPs and sylvatic mosquitoes such as *Haemagogus spp*. [10,11,12]. The existence of a sylvatic cycle in Africa was thereafter described [13]. This spillback from the urban to the sylvatic cycle in the Americas prevented the eradication of YFV in the continent, even after a continental *Ae. aegypti* eradication campaign and the availability of an efficient human vaccine [9,10,11]. Epizooties waves of YFV initiated in the north of South America, especially in the Amazon, have frequently caused outbreaks in Brazil and neighboring countries [14,15]. Accordingly, since 2014, a YFV spread from the Amazon toward the south and southeast Brazil was detected, which culminated in the largest and most severe sylvatic outbreak ever recorded in the country [16]. Therefore, as of 2016, YFV spread into the most populous Brazilian areas in the Southeast, having a low vaccination coverage, resulting in thousands of epizootics of NHPs and 2170 confirmed human cases and 932 deaths, mainly in 2017–2018 (Figure 1) [2,17]. Human cases had not been reported in most of the affected areas (the coastal Southeast) for almost 80 years, but the assessment of non-detected local enzootic sylvatic transmission in recent decades was lacking. In addition, the YFV sylvatic outbreak reached areas recording high *Ae. aegypti* house infestation indices, increasing the risk of urban transmission.

ZIKV was first isolated in 1947 from Rhesus monkeys used as sentinels in studies of sylvatic yellow fever in the Zika Forest in Uganda, Africa [6,18]. The virus has spread to several continents causing outbreaks in recent decades [18]. The first confirmation of the circulation of ZIKV in the Americas was made in northeast Brazil in 2015 [19] (Figure 1). In the same year, ZIKV reached the five Brazilian regions and other American countries. The outbreaks had dramatic consequences, such as association with cases of congenital microcephaly and other neurological disorders, and reports of non-vector transmission. The global dimension of the outbreaks led the World Health Organization (WHO) to declare Zika as a global sanitary emergency in 2016 [20]. Despite the problems in recognizing and notifying a new etiological agent, some authors have estimated 400.000 to 1.000.000 human Zika cases in 2015 [21]. Between 2016 and 2019, Brazil confirmed 253,221 Zika cases, peaking in 2015–2016 in the Southeast (Figure 1), as well as in most Brazilian regions. All Zika cases were of urban origin, where the mosquito *Ae. aegypti* was determined to be responsible for the vector transmissions [22]. 

Due to similarities in natural history between YFV and ZIKV, concerns have grown about the possibility of ZIKV establishing a sylvatic cycle in Brazil, which would prevent its eradication, as happened with YFV [4]. Although ZIKV has never been isolated from any other vertebrate besides humans in the Americas, and sylvatic NHP-biting mosquitoes have never been detected to be naturally infected with ZIKV, the findings of RNA fragments compatible with ZIKV and antibodies against this virus in synanthropic marmosets and capuchin monkeys captured in peri urban areas in Brazil, and the demonstration that marmosets can experimentally sustain viremia [23,24,25] have led to the hypothesis that NHPs would play a role in sustaining the urban transmission cycle or in establishing an sylvatic transmission cycle in the Americas. 

However, as far as we know, there are no published data on the investigations of ZIKV natural infection in wild free-living NHPs and mosquitoes. Dozens of other arboviruses have already been identified in Brazil, mainly in the Amazon forest [26] but, except for a few areas [27,28,29,30], little is known about the circulation of arboviruses in mosquitoes from the Atlantic forest of southeastern Brazil. 

In this context, we carried out extensive collections of free-living NHPs and mosquitoes before, during and after the recent ZIKV and YFV outbreaks (2015–2018) in both sylvatic and periurban areas in the state of Rio de Janeiro (RJ) and its borders in southeast Brazil, to explore the spatiotemporal circulation of these two viruses and test the hypothesis of their spillover/spillback between the urban and sylvatic cycles. As well as this, for the first time, we screened southeastern Atlantic forest mosquitoes for 35 other arboviruses species belonging to 94 different genotypes/serotypes. 

## 2. Materials and Methods 

### 2.1. Ethic Issues

Capture, biosafety and the handling of NHPs and mosquitoes were approved by the institutional Ethics Committee for Animal Experimentation (protocol CEUA/IOC-004/2015 - 10/04/2015, license L-037/2016 – 24/08/2016) and Brazilian Ministry of Environment (SISBIO 41837-3 - 20/05/2015, 52472-2 – 26/01/2016 and 54707-5 – 25/08/2016) and Rio de Janeiro’s Environment Agency (INEA 012/2016 - 07/02/2016 and 019/2018 - 19/04/2018).

### 2.2. Field Expeditions

**Figure 2:**
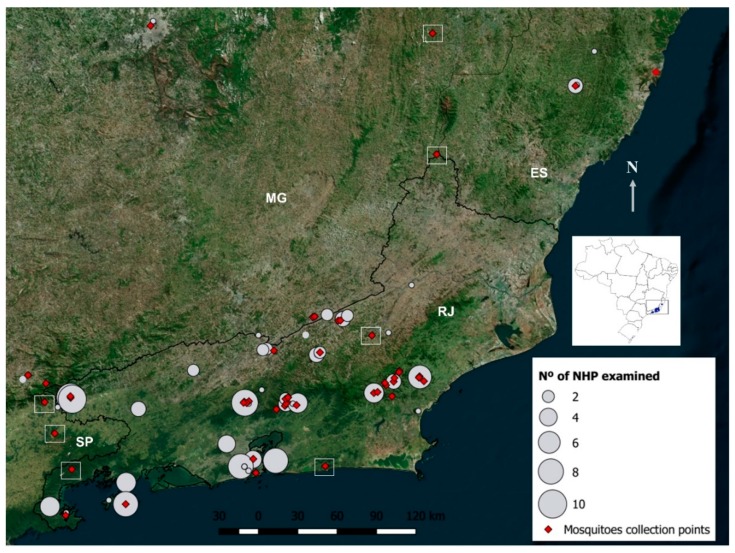
Satellite image showing mosquito and non-human primate (NHP) collection points. The number of NHPs captured is proportional to the size of the circle. Empty squares indicate areas where we attempt to capture NHPs without success.

Fieldwork was carried out from May 2015 to June 2018, comprising distinct epidemiological situations concerning ZIKV and YFV transmission in the southeast, that is before, during and after the ZIKV outbreak and before and during the YFV outbreak. We conducted short (1–5 days) and long (15 days) expeditions to catch mosquitoes and NHPs in the wild and periurban sites in RJ and its bordering states of Minas Gerais (MG), São Paulo (SP) and Espírito Santo (ES) (Figure 2). 

### 2.3. Non-Human Primate Samplings

NHP captures were performed using baited traps for small species, and mostly using anesthetic darts for larger ones [31,32]. A blood sample of 3–6 mL was collected from anesthetized animals. A subsample of ~500 µL of whole blood was immediately frozen in dry-ice or N_2_, and the remaining blood was left to coagulate. Following centrifugation (2000*g* × 10 min), sera were aliquoted and stored at −80 °C until use. Animals were released in the same capturing site after complete recovery from the anesthetic’s effects. Liver and/or blood samples were also collected from dying or recently dead NHPs, found by alerts from an information network we constructed [32].

### 2.4. Mosquito Collections

Mosquitoes were collected with entomological nets and manual aspirators indoors and outdoors, or with BG sentinel traps baited with CO_2_ installed along 300 m transects from the modified environment and forest edge to deep into the woods, as described in detail by Santos et al. (2018) [33]. In rural and forest areas, the collections were preferably done in the same areas the NHPs were captured (Figure 2). Caught mosquitoes were transported and identified at low temperature, pooled according to species, and stored at −80 °C until RNA extraction for viral diagnosis, as previously described [34,35].

### 2.5. Molecular Analyses

RNA was extracted from 140 μL of NHP serum, liver and/or whole blood using the Qiagen RNA Viral Kit, and from 140 μL of supernatant of mosquito pool homogenates using MACHEREY-NAGEL NucleoSpin 96 RNA extraction kits, following the manufacturer’s recommendations. For NHP samples, real-time RT-qPCR was performed in duplicate and in separate assays for YFV and ZIKV detection. For confirmation of any diagnosis, the amplicons obtained were directly sequenced without molecular cloning. The set of primers utilized in RT-qPCR and viral genome sequencing procedures followed previous reports [17,36]. 

Mosquito RNA were submitted to high-throughput, real-time PCR, developed by Moutailler et al. [35], to screen for 35 arboviruses of 94 different genotype/serotype (Table S1), including YFV and ZIKV. Briefly, specific primers and probes for the above-cited 35 mosquito-borne viruses, including 94 genotypes/serotypes targeted, were designed and validated on reference RNAs. Then, we used the BioMark Dynamic Arrays technology (Fluidigm Corporation, South San Francisco, CA, USA) for high-throughput, microfluidic, real-time PCR in plates of 96 x 96 which automatically cross, up to 96 samples with the 96 primers/probes sets, allowing the simultaneous screening as previously described [35]. 

### 2.6. Immunological Assays 

Plaque reduction neutralization test (PRNT): PRNTs were carried out on Vero cells in 24-well and 96-well plates for antibody neutralizing detections in all NHP serum samples against ZIKV and/or YFV, respectively [37]. Samples with PRNTs suspected to be positive for ZIKV or, exceptionally, against both YFV and ZIKV, were also tested by PRNT against DENV1-4 to check for cross-reaction among these flaviviruses. Briefly, serum samples were serially diluted from 1:5 to 640 (dilution factor = 2, to PRNT of YFV) and from 1∶10 to 1∶31,250 (dilution factor = 5, to PRNT of DENV and ZIKV), followed by the addition (volume/volume) of 120, 60 or 50 µL of DENV (1, 2, 3 or 4), ZIKV or YFV, corresponding to approximately 60 PFU / 200 µL (DENV), 100 PFU / 100 µL (ZIKV), 40 PFU / 50 µL (YFV), respectively. Then, plates were incubated at 37 °C in 5% CO_2_ for 1 h (neutralization step). For the adsorption step, 200 µL (PRNT of DENV) or 100 µL (PRNT of ZIKV) of mixture (serum + virus) were transferred to cell monolayers of 6-well or 24-well plates, previously prepared with 45 x 10^4^ cells/well or 20 x 10^4^ cells/well, respectively and incubated at 37 °C for 1 h. PRNT YFV was prepared in 96-well plates, then suspension of Vero cells (8 × 10^4^ cells/50 µL/well) was added into the mix (serum + virus) and plates were incubated at 37 °C for 3 h. After the adsorption step, media were discarded, and cell monolayers were overlaid with E199 medium, and incubated for 6–8 days at 37 °C in 5% CO_2_, until fixed with 10% formalin, and stained with crystal violet for plaques count. Neutralizing antibody titers were expressed by 90% of plaque reduction (PRNT_90_), and samples with titers > 10 were considered positive.

Enzyme Immunoassay (ELISA): ELISA was used for the determination of total IgG antibodies against the YFV, ZIKV and DENV of NHP samples with positive PRNT against one or more of these flaviviruses. The objective was to confirm diagnosis, as well as discriminate antiviral immune responses from the potential physical or chemical interference of any molecules and/or virucide factors in blood samples that may have affected the virus and/or reduced its adsorption to cellular monolayer in PRNT, as previously described [38], especially in some wild animal serum [27]. Briefly, the detection of the dengue IgG antibody was performed by a modification of Miagostovich et al. (1999) method [39]. For this, 100 μL of hyper-immune ascitic fluid, diluted in 0.1 M sodium carbonate buffer, pH 9.6, was added to each 96-well and plates were incubated overnight at 4 °C. After washing with PBS pH 7.4, wells were blocked by filling with standard diluent (PBS pH 7.4, 0.05% Tween, 3% normal goat serum) and incubated for 1 h at 37 °C. An antigen mix (DENV-1, 2, 3 and 4 samples) was applied to each well, following incubation for 1 h at 37 °C. Plates were washed again with PBS, and 100 μL of serum diluted 1:40 in Non-Fat Dry Milk (NFDM) diluent (PBS pH 7.4, 0.05% Tween, 3% non-fat dry milk) were added to each well. Plates were incubated for 1 h at 37 °C and washed. In total, 40 microliters of anti-human IgG conjugated to horseradish peroxidase diluted in NFDM diluent was added. After incubating for 1 h, plates were washed with PBS and 100 μL of substrate ABTS (2,2’-azino-di [3-etil-benthlazoline sulfonate]) and hydrogen peroxide were added to each well. Color development was continued at room temperature (RT) at an optical density of 405 nm, and the optical density of each dilution was subtracted from the corresponding dilution of each test sample. Index values > 0.150 were considered positive. 

For the detection of YFV IgG antibodies, 96-well plates were coated with 2.5 μg/mL of whole yellow fever virus particle diluted in coating buffer (carbonate–bicarbonate buffer, pH 9.6) and incubated overnight at 4 °C. In all washing steps, plates were rinsed mechanically five times with washing buffer (PBS pH 7.4 with 0.05% (v/v) of Tween-20 –PBS/T). Then, plates were blocked for 1 h at 37 °C with a blocking/diluent solution (BDS) (PBS/T, 0.05% (v/v) of BSA, 3% (w/v) of fetal bovine serum (FBS) and 5% (w/v) of skimmed milk). Serum samples were submitted to serial dilutions. An anti-YFV Serum, Monkey (YF - NIBSC) antibody was employed to derive a standard curve, in the range of 1 to 0.015 IU/mL. After 1 h at RT, plates were washed and incubated with the Anti-Monkey IgG peroxidase conjugated (A2054-Sigma), diluted 1:5000 in BDS, following incubation for 1 h at RT. After washing, 100 μL/well of substrate solution (TMB Plus^TM^ kem-en-tec) were added and after 15 min the reaction was stopped by adding stop-solution (2M H_2_SO_4_). The endpoint measurements were done at 450 nm and the absorbances of the serum sample dilutions were plotted on the standard curve. The results obtained by absorbance values >0.150 were calculated using the software SoftMax Pro^®^ by regression logistic for four parameters and the antibody titers were expressed in IU/mL. 

The Zika-Euroimmun commercial kit was used for ZIKV IgG antibody detection, following the manufacturer’s recommendations [40].

For all assays, serum of ZIKV-experimentally infected monkey, DENV-positive human, goat and healthy monkeys from different families (Callitrichidae, Cebidae and Atelidae), besides of YFV 17DD vaccinated monkeys were used as sensibility and specificity controls. 

## 3. Results

### 3.1. Molecular Findings

#### 3.1.1. Non-Human Primates

In total, 144 primates belonging to six neotropical species were captured in 27 counties in RJ and bordering southeastern states and tested by RT-qPCR for ZIKV and YFV detection. Two NHPs were examined before, 71 during, and 71 after the ZIKV outbreak, while 73 NHPs were tested before and 71 during the YFV outbreak in the Brazilian Southeast. 

**Figure 3:**
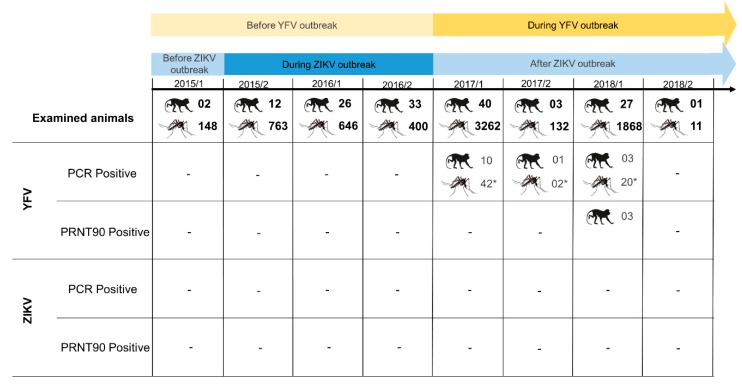
Number of examined and positive non-human primates and mosquitoes, according to epidemiological scenario, semester, year and diagnosis methods. Results of mosquito infections (*) are expressed in number of positive pools.

All animals consistently tested negative for ZIKV, regardless of collection site or epidemiological situation, that is before, during or after the ZIKV outbreak (Figure 1, Figure 2 and Figure 3; Table 1 and Table S2). On the other hand, 14 (9.7%) NHP samples were positive for YFV (CT values ranging from 10.4 to 16.8), all of which were collected during the YFV outbreak (from Jan/2017 to Jun/ 2018) and in counties where YFV circulation was suspected. Twelve out of these NHPs were howler monkeys—*Alouatta guariba clamitans*—and two were marmosets *Callithrix jacchus* x *Callithrix penicillata* hybrid. In total, 82% of howlers examined during the outbreak were positive. Eleven of the YFV-positive NHPs were collected in nine epizootic counties of RJ, while the other three were from a single county in ES. 

#### 3.1.2. Mosquitoes

A total of 5219 female mosquitoes belonging to 69 species were collected from the modified environment to deep into the forest of 19 counties, 13 in RJ, three in MG, two in ES and one in SP, undergoing a distinct epidemiological situation cornering ZIKV and YFV transmission (Figure 2; Table 1). Mosquitoes were grouped into 1298 pools and screened for the 94 genotypes and/or serotypes of arbovirus cited above, including YFV and ZIKV. 

All mosquitoes were negative for ZIKV, regardless of whether they originated from the wild of periurban and rural sites or were captured before, during or after the ZIKV outbreaks in the region (Figure 2 and Figure 3; Table 1). No other screened arbovirus was found in mosquitoes, except for YFV. The YFV mosquito infections revealed by the high-throughput PCR were confirmed by RNA sequencing and matched with those obtained when the homogenates of the same mosquito pools were screened by RT-qPCR using a distinct set of primers whose results were published elsewhere [34]. Infections by YFV were mostly found in the traditional vector *Haemagogus janthinomys,* as well as in *Haemagogus leucocelaenus,* while only one pool of *Sabethes chloropterus, Aedes scapularis* and *Aedes taeniorhynchus* were positives (Table 1). Viruses were successfully isolated in C6/36 cell culture from six pools of *Hg. janthinomys* and four of *Hg. leucocelaenus.*

The complete genome sequencing of YFV detected in NHPs and mosquitoes is available at GenBank (accession numbers MF423373, MF423374, MK333800, KY885000, KY885001, MF423375, MF423376, MF423377, MF423378, MF538785, MF538786) and confirmed the existence of a unique molecular signature of fixed amino acid mutations in highly conserved positions at NS3 and NS5 proteins in YFV, causing the current southeastern Brazilian outbreak [17,41].

### 3.2. Immunological Findings

In total, the sera of 118 out of 144 collected NHPs could be screened for neutralizing antibodies against YFV and ZIKV. The sera of 26 NHPs were not available, either because they were already found dead or consisted of small animals with low volaemia, such as young marmosets, preventing the collection of ideal amounts of blood.

None of the 73 NHPs captured before the YFV outbreak had neutralizing antibodies against YFV, while 4.2% (three out of 71 animals) captured during the outbreak were positive, all being capuchins (*Sapajus nigritus*) from the Itatiaia National Park, southern RJ (Figure 3, Table S2). We examined 12 capuchins from this park before the YFV outbreak and nine during the outbreak—of which eight were captured for the first time and one recaptured. A total of 37.5% (three of eight) were seropositive, and the recaptured one showed no seroconversion. 

Finally, we captured 14 marmosets in the edge of small forest fragments and modified environments inside the cities of Rio de Janeiro, Niterói and Belo Horizonte, where there was suspicion of YFV circulation, due to only one dead marmoset preliminarily diagnosed as due for YFV by the state surveillance system using PCR, but not confirmed by immunohistochemistry or RNA sequencing. Although captured in exactly the same site as where the epizootics were recorded, and belonging to the same family/group of dead animals, all 14 tested marmosets were negative for YFV neutralizing antibodies, showing no evidence of viral circulation in the suspected periurban and urban areas.

After testing NHP sera by PRNT and checking the results by ELISA, we concluded that none of them had specific neutralizing antibodies against ZIKV (Table S2, Table 2). Although the results of PRNT_90_ of 15 samples preliminarily suggested a protective response against ZIKV (Table 2), the combination of their results in PRNT-DENV, PRNT-YFV and ELISA for the detection of IgG against DENV, YFV, ZIKV confirmed cross-reactions and/or unspecific reactions. 

## 4. Discussion

YFV and ZIKV caused large outbreaks in Brazil from 2015 onwards, raising concern about the possibility of spillover from the sylvatic to the urban transmission cycle (in the case of the YFV) and spillback from the urban to an independent sylvatic cycle (for ZIKV). In the present work, after extensive sampling of both mosquito vectors and NHPs, we did not find evidence of spillover/spillback between urban and sylvatic cycles for these viruses.

Concerning the suspicion of YFV reurbanization, all urban or periurban mosquitoes and NHPs tested negative for YFV. Indeed, all vectors found infected herein and for previous authors were sylvatic species [34,42]. This finding, together with epidemiological, genetic and entomological records obtained during the outbreak [34,42,43,44,45,46], reinforced the sylvatic nature of the outbreak and the absence of spillover. Briefly, the demographic characteristics of the infected humans [43,46] and the spread rates of YFV over time and space and the genetic clades found in phylogeographic analyses were consistent with an NHP—sylvatic mosquitoes—human transmission [3,44,45,47,48]. Furthermore, all investigated cases also shared ecological conditions indicating their sylvatic origin, such as contact with forested areas, including people that entered into the jungle or live in the interface between cities and the natural environment [3,49,50]. 

YFV was not detected in any mosquito or NHPs captured prior to the outbreak. Moreover, the absence of protective antibodies against YFV in all NHPs examined before the outbreak suggests the absence of recent YFV circulation in southeastern brazil. Our results evidenced susceptible vertebrate hosts (NHP) of at least six species spread throughout RJ and its bordering states. This result also indicates that the territory was receptive to YFV transmission. In fact, the entire Atlantic coastal forest was considered a YFV-free area, without vaccination recommendation until the diagnosis of the first cases in early 2017 [15,16]. The large number of non-immune human and NHPs contributed to the rapid spread of the virus after its reintroduction. The occurrence and high frequency of competent vectors throughout the region [34] reinforced the receptivity and vulnerability of the region to YFV transmission. It seemed to be a matter of time. This scenario, combined with changes in the human behavioral patterns [3,49] and the potential role of distinct genetic characteristics of the circulating virus [17,41,47], may help to explain the magnitude of the outbreak. 

Howler monkeys (*Alouatta*) were the most affected genus by YFV in our sampling, as previously reported in Brazil and Argentina [51,52,53,54,55]. Even so, we did not find antibodies against YFV in the captured howlers, even in those animals tested during the outbreak. Probably, the majority of the exposed *Alouatta* were not able to produce enough protective antibodies and were rapidly killed by virus effects, as observed by Kumm and Laemmert, 1944 [56]. The same authors concluded that the difficulty of capturing these animals and the almost always fatal effect of some YFV strains on this NHP genus reduce the chances of finding immunized howler monkeys. In fact, Almeida et al. (2019) did not find any *Alouatta caraya* immunized in Rio Grande do Sul after the largest outbreak of YFV reached the region [51,57]. The encounter of non-immune howlers after the outbreak is worrisome because, although the size of the remaining populations is unknown, it could indicate that the Atlantic forest is still vulnerable to the circulation of YFV, as demonstrated by the recent finding of an infected *Alouatta g. clamitans* in the same area as the virus was detected in 2017 [58]. Continued surveillance and the vaccination campaigns in the region are imperative.

Unlike the howler monkeys, protective antibodies against YFV were detected in three *Sapajus nigritus* from the Itatiaia National Park, examined after confirmation of the YFV in the same reserve. Encountering immunized healthy animals against YFV with negative PCR, indicates that despite the susceptibility of some individuals, the immune response of this genus is more efficient in protection against the virus. Interestingly, immune capuchins were found after more than 10 years from confirmed YFV in humans in several regions of Brazil [56,59]. Due to their apparent higher resistance, longevity (reaching 40 years in laboratory) [60], abundance in several Brazilian cities and the relative ease of capture in some regions, we recommend that transverse immunological studies should be implemented in groups of *Sapajus sp*. with periodic recaptures for serological and molecular tests. These groups could be useful as sentinels, aiming at the early detection of viral circulation and human protection through the identification of priority areas for vaccination [61], mainly in areas with scant populations of howlers.

Marmosets are susceptible to YFV infections but seem to exhibit intermediate resistance to death when compared to capuchin and howler monkeys [62]. We found two individuals killed by the virus and many epizootics have been YFV-confirmed in the recent and previous outbreaks [55,63,64]. These findings raise concerns as many marmoset groups transit between forest fragments and cities, which could facilitate their role as a “bridge host” due to their behaviour, sometimes establishing close contact with humans [55,62]. Although we did not find marmosets with protective antibodies against YFV, longitudinal examinations of *Callithrix* groups may also be useful for YFV surveillance because they provide a shorter time cut than capuchin monkeys due to their shorter life cycle [31,56]. 

In relation to ZIKV, the combination of our analyzes (entomological, molecular and immunological) did not reveal evidence that an independent sylvatic cycle of this virus could have been established in RJ and the surroundings. Similar results were pointed out by Moreira-Soto et al. (2018), after analyzing 207 NHPs from two regions and three Brazilian states between 2012 and 2017 [38]. The authors did not detect ZIKV through RT-qPCR and reported to have found specific antibodies in six NHPs, with low titers, even though they were collected in urban and periurban areas in some of the regions most affected by the outbreak [38]. On the other hand, after performing NHP captures in a peridomestic environment contiguous to houses whose inhabitants had Zika, Favoretto et al. (2019) reported to have detected ZIKV genome in nine out of 132 marmosets or capuchin monkeys, captured mainly in 2015 [24]. A genome compatible to ZIKV was also detected in the viscera of urban and periurban marmosets and capuchins under suspicion of yellow fever infection, found dead in SP and MG [23]. Despite the generally high CTs and the discordant results obtained from different viscera of the same animal, the authors claimed to have detected the presence of the virus in 32 (39%) of the 82 tested NHPs [23]. The same authors also detected ZIKV in the domestic mosquitoes *Ae. aegypti*, the species implicated in the urban transmission in Brazil [22]. Although the evidence of ZIKV circulation among urban or periurban NHPs and mosquitoes is a concern, it does not directly imply the establishment of an independent sylvatic cycle, maintained between wild mosquitoes and NHPs. 

Although we found 15 suggestive responses in ZIKV-PRNT_90_, almost all were detected in hemolyzed serum and cross-reacted with other viruses when screened by PRNT-YFV, PRNT-DENV1-4 and/or ELISA for DENV, YFV and ZIKV. Notably, samples from monkeys that died of yellow fever [six out of the 15 (RJ87, RJ95, RJ96, RJ104, ES04, ES05)] whose blood was obtained from postmortem cardiac puncture, had a viscous, greenish appearance. The PRNT of serum samples with these aspects was suggested to have high antibody titles against all tested viruses (Table 2). The same was observed with the serum of a howler (RJ46) that was attacked by bees at the time of capture and died of anaphylaxis; its blood was collected before death. Therefore, we suspected that the PRNT results of these specific animals were consequences of non-specific virucidal agents, inactivating or reducing viruses’ adsorption capacity in the cellular monolayer. In addition to the already known cross-reaction between *Flavivirus* [24,57,61], this non-specific agent explains the high degree of cross-reactivity with the other virus tested, especially in the hemolyzed samples. This hypothesis was confirmed by ELISA assays, which did not recognize specific immunoglobulins in any of the 13 available sera against any of the tested viruses (ZIKV, DENV, YFV) (Table 2). It is known that serum quality, especially the degree of hemolysis, directly influences serological tests [65,66]. Nevertheless, we decided to test all the samples, including the hemolyzed, because they were the only available material obtained from wild animals that rarely can be examined. In this way, the ELISA test is a useful tool when aiming to resolve doubts about specific or nonspecific PRNT responses, as demonstrated in few of our results. 

From an entomological point of view, the evidence of ZIKV-sylvatic transmission was null. All captured mosquitoes tested negative for this and other tested arboviruses, except YFV. Noteworthy, during a ZIKV outbreak in Guadeloupe and French Guiana, we succeed in detecting ZIKV in urban mosquitoes with the same high throughput system [35]. Sylvatic Neotropical mosquitoes belonging to six species and three genera that have been experimentally challenged with three ZIKV isolates thus far were refractory or exhibited low infection rates [67,68]. Therefore, considering the low vectorial competence of sylvatic mosquitoes tested thus far, coupled with the short, low and asymptomatic viremia of experimentally injected NHPs [69,70], and the fact that recent evidence has concentrated their findings in urban or periurban environments, we assume that there are still no reasons to suppose that ZIKV has established an independent sylvatic cycle in the forests of the RJ and surroundings in Southeast Brazil. Further studies, especially experimental infections of NHP and systematic collections of NHPs for molecular and serological surveillance should be conducted in order to shed more light on this issue. 

Finally, it is important to emphasize the importance of combining arbovirus diagnosis techniques, especially during outbreaks when surveillance becomes more sensitive and a very large number of samples arrive at the laboratories. The combination of serological, immunohistochemical and molecular tests is extremely useful for retelling the history and/or confirming the infection of a pathogen. For example, we provided evidence that the marmosets (*Callithrix sp*.) whose cause mortis was diagnosed as YFV by RT-PCR in three large Brazilian cities may have been false positives, since other finds, such as serology of the other members of the group, allied to the entomological, histopathological and epidemiological results, did not support the molecular diagnosis. The same occurred with some suspicions of human urban yellow fever cases, which were later proven to have been of sylvatic origin [50].

In summary, the present study showed that there is no evidence that the ZIKV established an independent sylvatic cycle in RJ, and provided new evidence that there was no urban transmission of YFV in southeast Brazil during the current outbreak [34,44]. However, in view of the receptivity of the state, verified by the low prevalence of antibodies in NHPs examined during the outbreak, we recommend the strengthening of surveillance. Immunological and molecular techniques associated with the monitoring of NHP (mainly *Sapajus sp.*) and wild mosquitoes’ populations should be implemented in order to detect the early circulation of arboviruses that could threaten humans.

## Figures and Tables

**Table 1:** Mosquito species collected and screened through 94 arbovirus genotypes in four Brazilian states: Espírito Santo (ES), Minas Gerais (MG), Rio de Janeiro (RJ) and São Paulo (SP).

Species Per Year / Semester	Number	Date	Counties (State)	Result PCR*^a^*
*Aedes albopictus*	4	2015/1	**Macaé (RJ)**	Neg.
*Aedes scapularis*	12	2015/1		Neg.
*Culex sp.*	1	2015/1		Neg.
*Haemagogus leucocelaenus*	17	2015/1		Neg.
*Limatus durhamii*	2	2015/1		Neg.
*Psorophora ferox*	10	2015/1		Neg.
*Psorophora sp.*	5	2015/1		Neg.
*Runchomyia humboldti*	1	2015/1		Neg.
*Runchomyia reversa / theobaldi*	1	2015/1		Neg.
*Sabethes albiprivus*	1	2015/1		Neg.
*Wyeomyia aporonoma/staminifera*	1	2015/1		Neg.
*Wyeomyia (Pho.) sp.*	89	2015/1		Neg.
*Wyeomyia sp.*	4	2015/1		Neg.
**2015/1**	**148**			Neg.
*Aedes albopictus*	14	2015/2	**Guapimirim, Macaé, Magé, Miguel Pereira, Teresópolis (RJ)**	Neg.
*Aedes fulvithorax*	1	2015/2		Neg.
*Aedes scapularis*	94	2015/2		Neg.
*Aedes serratus*	1	2015/2		Neg.
*Aedes terrens*	25	2015/2		Neg.
*Anopheles cruzii*	1	2015/2		Neg.
*Anopheles sp.*	3	2015/2		Neg.
*Culex sp.*	9	2015/2		Neg.
*Culex nigripalpus*	10	2015/2		Neg.
*Haemagogus janthinomys*	60	2015/2		Neg.
*Haemagogus leucocelaenus*	87	2015/2		Neg.
*Limatus durhamii*	26	2015/2		Neg.
*Limatus pseudomethisticus*	2	2015/2		Neg.
*Onirion personatum*	41	2015/2		Neg.
*Psorophora ferox*	21	2015/2		Neg.
*Psorophora sp.*	3	2015/2		Neg.
*Runchomyia cerqueirai*	15	2015/2		Neg.
*Runchomyia frontosa*	8	2015/2		Neg.
*Runchomyia humboldti*	48	2015/2		Neg.
*Runchomyia reversa / theobaldi*	1	2015/2		Neg.
*Runchomyia sp.*	20	2015/2		Neg.
*Sabethes albiprivus*	2	2015/2		Neg.
*Sabethes aurescens*	19	2015/2		Neg.
*Sabethes chloropterus*	1	2015/2		Neg.
*Sabethes chloropterus’*	5	2015/2		Neg.
*Sabethes fabricii’*	2	2015/2		Neg.
*Sabethes identicus*	1	2015/2		Neg.
*Sabethes intermedius*	2	2015/2		Neg.
*Sabethes melanonymphe*	7	2015/2		Neg.
*Sabethes xyphides*	2	2015/2		Neg.
*Sabethes sp.*	8	2015/2		Neg.
*Sh. fluviatilis*	49	2015/2		Neg.
*Shanonniana sp*	28	2015/2		Neg.
*Trichoprosopon digitatum*	24	2015/2		Neg.
*Trichoprosopon pallidiventer*	5	2015/2		Neg.
*Trichoprosopon sp.*	1	2015/2		Neg.
*Wyeomyia aporonoma/staminifera*	5	2015/2		Neg.
*Wyeomyia bonnei/deanei*	1	2015/2		Neg.
*Wyeomyia davisi*	11	2015/2		Neg.
*Wyeomyia mystes*	5	2015/2		Neg.
*Wyeomyia pilicauda*	42	2015/2		Neg.
*Wyeomyia theobaldi*	4	2015/2		Neg.
*Wyeomyia (Pho.) sp.*	43	2015/2		Neg.
*Wyeomyia sp.*	6	2015/2		Neg.
**2015/2**	**763**			Neg.
*Aedes albopictus*	1	2016/1	**Guapimirim, Macaé, Miguel Pereira, Nova Friburgo (RJ)**	Neg.
*Aedes scapularis*	56	2016/1		Neg.
*Aedes serratus*	2	2016/1		Neg.
*Aedes terrens*	44	2016/1		Neg.
*Anopheles bellator*	2	2016/1		Neg.
*Anopheles cruzii*	12	2016/1		Neg.
*An. hominales*	1	2016/1		Neg.
*Anopheles lutzi*	1	2016/1		Neg.
*Anopheles sp.*	14	2016/1		Neg.
*Culex sp.*	44	2016/1		Neg.
*Haemagogus janthinomys*	7	2016/1		Neg.
*Haemagogus leucocelaenus*	10	2016/1		Neg.
*Limatus durhamii*	31	2016/1		Neg.
*Limatus pseudomethisticus*	12	2016/1		Neg.
*Onirion personatum*	30	2016/1		Neg.
*Psorophora ferox*	3	2016/1		Neg.
*Runchomyia cerqueirai*	1	2016/1		Neg.
*Runchomyia frontosa*	22	2016/1		Neg.
*Runchomyia humboldti*	25	2016/1		Neg.
*Runchomyia sp.*	29	2016/1		Neg.
*Sabethes aurescens*	4	2016/1		Neg.
*Sabethes identicus*	3	2016/1		Neg.
*Sabethes melanonymphe*	6	2016/1		Neg.
*Sabethes sp.*	19	2016/1		Neg.
*Sabethini*	5	2016/1		Neg.
*Shannoniana fluviatilis*	84	2016/1		Neg.
*Trichoprosopon digitatum*	4	2016/1		Neg.
*Trichoprosopon pallidiventer*	18	2016/1		Neg.
*Wyeomyia aporonoma/staminifera*	5	2016/1		Neg.
*Wyeomyia bonnei/deanei*	4	2016/1		Neg.
*Wyeomyia cerqueirai*	1	2016/1		Neg.
*Wyeomyia confusa*	1	2016/1		Neg.
*Wyeomyia davisi*	1	2016/1		Neg.
*Wyeomyia pallidoventer*	6	2016/1		Neg.
*Wyeomyia palmata/galvoi*	3	2016/1		Neg.
*Wyeomyia pilicauda*	8	2016/1		Neg.
*Wyeomyia (Pho.) sp.*	118	2016/1		Neg.
*Wyeomyia sp.*	9	2016/1		Neg.
**2016/1**	**646**			Neg.
*Aedes aegypti*	9	2016/2	**Itamonte (MG); Queluz (SP); Itatiaia, Miguel Pereira, Rio de Janeiro, Sumidouro, Teresópolis (RJ)**	Neg.
*Aedes fluviatilis*	1	2016/2		Neg.
*Aedes scapularis*	11	2016/2		Neg.
*Aedes terrens*	4	2016/2		Neg.
*Anopheles cruzii*	4	2016/2		Neg.
*Anopheles sp.*	13	2016/2		Neg.
*Culex sp.*	1	2016/2		Neg.
*Haemagogus janthinomys*	8	2016/2		Neg.
*Haemagogus leucocelaenus*	22	2016/2		Neg.
*Limatus durhamii*	34	2016/2		Neg.
*Limatus pseudomethisticus*	42	2016/2		Neg.
*Onirion personatum*	8	2016/2		Neg.
*Psorophora ferox*	1	2016/2		Neg.
*Runchomyia frontosa*	7	2016/2		Neg.
*Runchomyia humboldti*	3	2016/2		Neg.
*Runchomyia sp.*	13	2016/2		Neg.
*Sabethes albiprivus*	4	2016/2		Neg.
*Sabethes aurescens*	3	2016/2		Neg.
*Sabethes auresces*	2	2016/2		Neg.
*Sabethes intermedius*	3	2016/2		Neg.
*Sabethes melanonymphe*	3	2016/2		Neg.
*Sabethes sp.*	26	2016/2		Neg.
*Shannoniana fluviatilis*	18	2016/2		Neg.
*Trichoprosopon castroi/similis*	9	2016/2		Neg.
*Trichoprosopon digitatum*	6	2016/2		Neg.
*Trichoprosopon pallidiventer*	18	2016/2		Neg.
*Wyeomyia antunesi*	3	2016/2		Neg.
*Wyeomyia aporonoma/staminifera*	1	2016/2		Neg.
*Wyeomyia confusa*	60	2016/2		Neg.
*Wyeomyia davisi*	5	2016/2		Neg.
*Wyeomyia exallos*	1	2016/2		Neg.
*Wyeomyia incaudata*	2	2016/2		Neg.
*Wyeomyia longirostris*	2	2016/2		Neg.
*Wyeomyia lutzi*	4	2016/2		Neg.
*Wyeomyia palmata/galvoi*	6	2016/2		Neg.
*Wyeomyia pilicauda*	11	2016/2		Neg.
*Wyeomyia (Pho.) sp.*	23	2016/2		Neg.
*Wyeomyia sp.*	9	2016/2		Neg.
**2016/2**	**400**			Neg.
*Aedes aegypti*	94	2017/1	**Belo Horizonte, Simonésia (MG); Domingos Martins, Serra (ES); Angra dos Reis, Casimiro de Abreu, Macaé, Maricá, Petrópolis, Rio de Janeiro (RJ)**	Neg
*Aedes albopictus*	1	2017/1		Neg.
*Aedes argyrothorax*	2	2017/1		Neg.
*Aedes fulvithorax*	2	2017/1		Neg.
*Aedes rhyacophilus*	1	2017/1		Neg.
*Aedes scapularis*	876	2017/1		YFV
*Aedes serratus*	9	2017/1		Neg.
*Aedes taeniorhynchus*	892	2017/1		YFV
*Aedes terrens*	7	2017/1		Neg.
*Aedes sp.*	22	2017/1		Neg.
*Anopheles sp.*	4	2017/1		Neg.
*Coquillettidia sp.*	72	2017/1		Neg.
*Coquillettidia albicosta*	1	2017/1		Neg.
*Coquillettidia hermanoi*	1	2017/1		Neg.
*Coquillettidia justamansonia*	16	2017/1		Neg.
*Coquillettidia nigricans*	6	2017/1		Neg.
*Coquillettidia shannoni*	1	2017/1		Neg.
*Coquillettidia venezuelensis*	2	2017/1		Neg.
*Culex grupo Coronata*	1	2017/1		Neg.
*Culex sp.*	56	2017/1		Neg.
*Culex declarator*	4	2017/1		Neg.
*Culex nigripalpus*	103	2017/1		Neg.
*Haemagogus janthinomys*	8	2017/1		YFV
*Haemagogus leucocelaenus*	199	2017/1		YFV
*Limatus durhamii*	189	2017/1		Neg.
*Limatus pseudomethisticus*	2	2017/1		Neg.
*Limatus sp.*	12	2017/1		Neg.
*Mansonia indubitans*	75	2017/1		Neg.
*Mansonia titillans*	14	2017/1		Neg.
*Mansonia sp.*	10	2017/1		Neg.
*Psorophora ferox*	21	2017/1		Neg.
*Psorophora lutzii/amazonica*	4	2017/1		Neg.
*Psorophora sp.*	10	2017/1		Neg.
*Runchomyia frontosa*	13	2017/1		Neg.
*Runchomyia humboldti*	12	2017/1		Neg.
*Runchomyia sp.*	10	2017/1		Neg.
*Sabethes petrocchiae*	60	2017/1		Neg.
*Sabethes albiprivus*	194	2017/1		Neg.
*Sabethes aurescens*	2	2017/1		Neg.
*Sabethes chloropterus*	4	2017/1		YFV
*Sabethes fabricii’*	3	2017/1		Neg.
*Sabethes quasicyaneus*	1	2017/1		Neg.
*Sabethes whitmani*	1	2017/1		Neg.
*Sabethes sp.*	47	2017/1		Neg.
*Shannoniana fluviatilis*	2	2017/1		Neg.
*Trichoprosopon digitatum*	1	2017/1		Neg.
*Trichoprosopon pallidiventer*	7	2017/1		Neg.
*Trichoprosopon sp.*	3	2017/1		Neg.
*Wyeomyia aporonoma/staminifera*	7	2017/1		Neg.
*Wyeomyia bourrouli/ forcipenis*	11	2017/1		Neg.
*Wyeomyia confusa*	40	2017/1		Neg.
*Wyeomyia edwardsi*	9	2017/1		Neg.
*Wyeomyia incaudata*	7	2017/1		Neg.
*Wyeomyia medioalbipes*	14	2017/1		Neg.
*Wyeomyia melanocephala’*	1	2017/1		Neg.
*Wyeomyia mystes*	9	2017/1		Neg.
*Wyeomyia palmata/galvoi*	17	2017/1		Neg.
*Wyeomyia pilicauda*	4	2017/1		Neg.
*Wyeomyia (Mia.) sp.*	1	2017/1		Neg.
*Wyeomyia (Pho.) sp.*	20	2017/1		Neg.
*Wyeomyia sp.*	45	2017/1		Neg.
**2017/1**	**3262**			
**TOTAL**	**5219**	**2015–2017**	**19**	**YFV**

*a*: The YFV mosquito infections revealed by the high throughput PCR were confirmed by RNA sequencing and RT-qPCR using distinct set of primers, whose results were published elsewhere [34,35]. 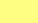 Mosquitoes captured before ZIKV and YFV outbreaks. 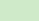 Mosquitoes examined during the ZIKV and before YFV outbreaks. 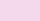 Mosquitoes examined after ZIKV and during YFV outbreaks

**Table 2:** List of samples that showed unspecific response during the PRNT_90_ assays for tested viruses.

Non-Human Primate Data				Molecular Results		PRNT Results							ELISA Results			Conclusions	
Code	Species	State	Health state	rt-PCR	CT	YFV 90%	ZIKV 90%	DENV1 90%	DENV2 90%	DENV3 90%	DENV 4 90%	Serum Quality	YFV	ZIKV	DENV	Imunological	Molecular
RJ10	Callithrix jacchus*	RJ	Healthy	Neg.	_	< 5	168.3	28.9	23.9	48.0	< 10	Hemoly zed++	Neg.	Neg.	Neg.	Negative	Negative
RJ18	Callithrix jacchus*	RJ	Healthy	Neg.	_	< 5	17.2	<20	< 10	< 10	< 10	Hemoly zed++	Neg.	Neg.	Neg.	Negative	Negative
RJ46	*Alouatta g. clamitans*	MG	Dying	Neg.	_	29.9	341.8	180.0	<10	< 10	< 10	Hemoly zed+++	Neg.	Neg.	Neg.	Negative	Negative
RJ60	*Leontopithecus rosalia*	RJ	Healthy	Neg.	_	<10	10.8	-	< 10	< 10	< 10	Hemoly zed++	Neg.	Neg.	Neg.	Negative	Negative
RJ62	*Leontopithecus rosalia*	RJ	Healthy	Neg.	_	< 5	41.6	<100	< 10	< 10	< 10	Hemoly zed++	-	-	-	Negative	Negative
RJ64	*Leontopithecus rosalia*	RJ	Healthy	Neg.	_	< 5	13.1	<100	14.4	< 10	< 10	Hemoly zed++	-	-	-	Negative	Negative
RJ87	*Alouatta g. clamitans*	RJ	Dead	YFV	Conv.	<20	87.1	<500	< 10	29.5	< 10	Hemoly zed+++	Neg.	Neg.	Neg.	Negative	YFV
RJ91B	Callithrix jacchus*	RJ	Healthy	Neg.	_	< 5	31.26	<20	-	< 10	< 10	Hemoly zed++	Neg.	Neg.	Neg.	Negative	Negative
RJ95	*Alouatta g. clamitans*	RJ	Dead	YFV	11.7	< 5	65.9	30.0	10.3	< 10	< 10	Hemoly zed+++	Neg.	Neg.	Neg.	Negative	YFV
RJ96	*Alouatta g. clamitans*	RJ	Dead	YFV	Conv.	<40	420.3	-	>250	< 10	< 10	Hemoly zed+++	Neg.	Neg.	Neg.	Negative	YFV
RJ104	Callithrix jacchus*	RJ	Dead	YFV	13.7	< 5	11.6	-	-	-	-	Hemoly zed+	Neg.	Neg.	Neg.	Negative	YFV
AR03	*Alouatta g. clamitans*	RJ	Healthy	Neg.	_	< 5	12.7	-	-	-	-	Hemoly zed++	Neg.	Neg.	Neg.	Negative	Negative
RJ118	Callithrix jacchus*	RJ	Healthy	Neg.	_	< 5	39.1	-	-	-	-	Hemoly zed+++	Neg.	Neg.	Neg.	Negative	Negative
ES01	*Brachyteles arachnoides*	ES	Healthy	Neg.	_	< 5	14.5	<10	< 10	17.17	< 10	Normal	Neg.	Neg.	Neg.	Negative	Negative
ES04	*Alouatta g. clamitans*	ES	Dying	YFV	Conv.	6.9	85.4	-	24.51	< 10	< 10	Hemoly zed+++	Neg.	Neg.	Neg.	Negative	YFV

“-“ means that it was not possible to perform the test due to sample exhaustion; + : indicate the degree of hemolysis; conv: conventional RT-PCR; *: hybrids of *C. jacchus* and *C. penicillata*. 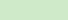 NHPs examined during Zika (ZIKV) and before Yellow Fever (YFV) outbreaks. 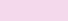 NHPs examined after ZIKV and during YFV outbreaks.

## Supplementary Materials

The following are available online at https://www.mdpi.com/1999-4915/12/4/364/s1, Table S1: List of viruses and serotypes tested by high throughput real time PCR, Table S2: Summary of the characteristics of non-human primates examined.
