# Supplementary material for: Survey on Non-Human Primates and Mosquitoes Does not Provide Evidences of Spillover/Spillback between the Urban and Sylvatic Cycles of Yellow Fever and Zika Viruses Following Severe Outbreaks in Southeast Brazil
Source: Viruses. 2020 Mar 26;12(4):364. doi: 10.3390/v12040364 (PMC7232473; doi:10.3390/v12040364)
Supplement: Supplementary file 1 [file viruses-12-00364-s001.zip › viruses-689607.suppl zip/Table S1.pdf]

Table S1: List of viruses and serotypes tested by high throughput real time PCR

**Bussuquara**
**Cache Valley**
**California encephalitis 1**
**California encephalitis 2**
**Chikungunya**
**Chikungunya_ECSA**
**Chikungunya Asia**
**Chikungunya IndECSA**
**Chikungunya Wafr**
**Dengue 1**
**Dengue 2**
**Dengue 3**
**Dengue 4**
**East equine encephalitis 1**
**East equine encephalitis 2**
**West equine encephalitis B1**
**West equine encephalitis B2**
**Venezuelan equine encephalitis 1**
**Venezuelan equine encephalitis 2**
**Venezuelan equine encephalitis 3**
**Venezuelan equine encephalitis 4**
**Venezuelan equine encephalitis 5**
**Venezuelan equine encephalitis 6**
**Venezuelan equine encephalitis 7**
**Venezuelan equine encephalitis 8**
**Venezuelan equine encephalitis 9**
**Venezuelan equine encephalitis 10**
**Venezuelan equine encephalitis 11**
**Venezuelan equine encephalitis 12**
**Venezuelan equine encephalitis 13**
**Group C**
Apeu
Caraparu 1
Caraparu 2

**Guama 1**
**Guama 2**
**Guama 3**
**Guama 4**
**Guama 5**
**Guaroa S1**
**Guaroa S2**
**Guaroa S3**
**Ilhéus**
**Jamestown Canyon**
**Jurona**
**Keystone**
**La crosse 1**
**La crosse 2**
**La crosse 3**
**La crosse 4**
**La crosse 5**
**La crosse 6**
**La crosse 7**
**La crosse 8**
**Mayaro 1**
**Mayaro 2**
**Oropouche**
**Rocio**
**Sindbis 2-3**
**Sindbis 1**
**Sindbis 4**
**Sindbis 5**
**Sindbis 6**
**Snowshoe Hare**
**Saint Louis**
**Tacaiuma**
**Trivittatus**
**Una 1**

**West Nile 2.1**
**West Nile 2.2**
**West Nile 3**
**West Nile 4**
**Yellow Fever AC_AE**
**Yellow Fever SA1**
**Yellow Fever SA2**
**Yellow Fever WA1**
**Yellow Fever WA3**
**Yellow Fever WA4**
**Zika 2**
**Zika 4**
**Zika 6**
**Zika 7**
**Zika 8**

Itaqui
Marituba
Murutucu
Nepuyo
Oriboca
Restan

**Una 2**
**Una 3**
**Una 4**
**West Nile 1A**
**West Nile 1B**
**West Nile 1C**
